# Automatic Cyclic Alternating Pattern (CAP) analysis: Local and multi-trace approaches

**DOI:** 10.1371/journal.pone.0260984

**Published:** 2021-12-02

**Authors:** Maria Paola Tramonti Fantozzi, Ugo Faraguna, Adrien Ugon, Gastone Ciuti, Andrea Pinna

**Affiliations:** 1 Laboratoire d’Informatique de Paris 6, CNRS, Sorbonne Université, Paris, France; 2 The BioRobotics Institute, Scuola Superiore Sant’Anna, Pontedera, Italy; 3 Department of Translational Research and of New Surgical and Medical Technologies, University of Pisa, Pisa, Italy; 4 Department of Developmental Neuroscience, IRCCS Fondazione Stella Maris, Pisa, Italy; 5 ESIEE-Paris, Cité Descartes, Noisy-le-Grand, France; IRCCS Istituto Delle Scienze Neurologiche di Bologna, ITALY

## Abstract

The Cyclic Alternating Pattern (CAP) is composed of cycles of two different electroencephalographic features: an activation A-phase followed by a B-phase representing the background activity. CAP is considered a physiological marker of sleep instability. Despite its informative nature, the clinical applications remain limited as CAP analysis is a time-consuming activity. In order to overcome this limit, several automatic detection methods were recently developed. In this paper, two new dimensions were investigated in the attempt to optimize novel, efficient and automatic detection algorithms: 1) many electroencephalographic leads were compared to identify the best local performance, and 2) the global contribution of the concurrent detection across several derivations to CAP identification. The developed algorithms were tested on 41 polysomnographic recordings from normal (n = 8) and pathological (n = 33) subjects. In comparison with the visual CAP analysis as the gold standard, the performance of each algorithm was evaluated. Locally, the detection on the F4-C4 derivation showed the best performance in comparison with all other leads, providing practical suggestions of electrode montage when a lean and minimally invasive approach is preferable. A further improvement in the detection was achieved by a multi-trace method, the Global Analysis—Common Events, to be applied when several recording derivations are available. Moreover, CAP time and CAP rate obtained with these algorithms positively correlated with the ones identified by the scorer. These preliminary findings support efficient automated ways for the evaluation of the sleep instability, generalizable to both normal and pathological subjects affected by different sleep disorders.

## Introduction

The Cyclic Alternating Pattern (CAP) has been established as a marker of sleep instability [1, 2]. It is a recurring physiologic event usually occurring during the non-rapid eye movement (NREM) sleep stages. CAP events are composed of transient periods of high amplitude electrocortical phasic phases (A phases) interrupting the background activities (B phases). A CAP cycle is composed of a phase A and the following phase B and at least two consecutive CAP cycles define a CAP sequence. All CAP sequences begin with a phase A and end with a phase B and the duration of each phase is 2–60 s [1]. Differently to the CAP B phases, characterized by attenuated autonomic and muscular activities, CAP A phases are generally related to different degrees of increased cardiorespiratory activities and muscle tones and can be classified into three different subtypes [3]:

- A1, prevalent in the descending branch of the sleep cycles (from light to deep NREM sleep), is characterized by slow frequency, high voltage electroencephalographic (EEG) synchronized patterns associated with weak modification of the muscle tone and the cardiorespiratory rates. If present, the EEG desynchronized activity occupies less than 20% of the entire phase A duration.
- A3, prevalent in the ascending branch of the sleep cycle (from deep to light sleep), is characterized by rapid and relatively low amplitude EEG activities, with a strong increase of the muscle tone and the cardiorespiratory rates. The EEG desynchronized activity occupies more than 50% of the total phase A time.
- A2 represents a mixed phase between A1 and A3 with 20–50% of the total A phase time occupied by desynchronized EEG with predominance in the ascending branch of the sleep cycle.

The amplitude of the A phases is almost 1/3 higher than the background voltage [1] and a topographic analysis shows a clear prevalence over the frontal/parietal-occipital areas of the A1 and A3 CAP A subtypes, respectively, with a peak in the midline and symmetrical spreading over the two hemispheres [4].

The sum of all sleep time including CAP sequences is described as CAP time. The ratio of CAP time to total sleep time represents the so-called CAP rate [3], the most reliable metrics of the sleep arousal instability [2]. This latter parameter is useful to extract relevant information for subject’s sleep quality evaluation: the hypnogram is a useful tool to evaluate the sleep macro-architecture, highlighting the presence of sleep disorders, but it is not sufficient to evaluate the sleep micro- architecture in order to identify sleep continuity on a finer scale.

Indeed, CAP rate increases when sleep is disturbed by internal or external factors of perturbation [5], and its variations reflect the subjective appreciation of sleep quality: higher values of CAP rate are related to poorer sleep quality [3].

Several physiological and clinical conditions are associated with CAP-related change: in school-age children as well as in adults, CAP rate tends to physiologically increase across development and aging, with a concurrent decrease in CAP A1 percentage and an increase in A2 and A3 [2, 6, 7]. Moreover, CAP rate is increased also in conditions of sleep perturbation, such as insomnia [8–10], depression [11], periodic limb movements [12] and nocturnal frontal lobe epilepsy [13]. Also, in severe obstructive sleep apnea syndrome (OSAS), CAP rate increases, alongside a relative increase in subtypes A3 percentages at the expense of a reduction of subtypes A1. These CAP-changes are reversible after continuous positive airway pressure (CPAP) treatment in OSAS subjects [14], administration of some hypnotic medication in insomniacs [8, 9, 15, 16] recovery sleep time after previous sleep deprivation [17, 18]. Although its value in accurately describing sleep instability, CAP analysis did not fully enter the clinical practice. One of the main limitations is the time-consuming and tedious activity implied by this approach, since specialists in sleep medicine with specific skills and knowledge have to visually identify each A phase (with its B phase associated) present in the whole night EEG signal [19–21]. Moreover, it has been estimated that the CAP inter-scorer agreement ranges from 69 to 77% [22]. In the perspective of practical clinical utilization several authors have shared the urge of developing efficient automated EEG methods [21].

In this direction several attempts have been made to implement automatic methods for the CAP detection [20, 23–29]. In general, time and frequency domain analysis allowed the identification of the EEG features, characteristic of the CAP A phases. The first automatic algorithms were based on EEG band-related descriptors or activity indices, representing the normalized instantaneous amplitude or power over its background activity. This ratio is then compared with specific thresholds for the detection and classification of CAP A phases [20, 23–25, 30, 31]. Although these simple approaches proved rather accurate in the detection of CAP, they were applied to a limited number of EEG signals of mainly healthy subjects [20, 23–25, 30]. More recently, entropy based features were exploited by Karimzadeh and colleagues [32] to identify CAP A phases from the background activity, using the k-nearest neighbours (kNN), the linear discriminant analysis (LDA) and the support vector machine (SVM) as classifiers. This latter machine learning method was investigated for the CAP A automatic detection also by Mariani and colleagues [33] achieving performance metrics similar to those obtained with artificial neural network [26] and adaptive reservoir genetic algorithm [34]. Table 1 summarizes the Sensitivity and the Accuracy performance metrics highlighted in some of the articles cited above.

**Table 1:** Performance metrics in the articles citated.

CAP A phases identification—Method	Reference	Sensitivity (%)	Accuracy (%)
Band Descriptor	[23]	89.8	89.8
	[24]	84	77
	[30]	69.55	87.19
Artificial Neural Network	[26]	75.65	81.55
Support Vector Machines	[33]	73.82	84.05
Adaptive Reservoir Genetic Algorithm	[34]	85.7	-

Inspired by the most accurate band descriptors-based algorithms, Ferri and colleagues developed a simple efficient automatic and parametric method for the analysis of NREM sleep instability, useful in clinical application, focusing their attention on the C4-A1 EEG monopolar derivation of healthy subjects [29]. This approach, providing highly significant correlations between the visually extracted CAP parameters and the NREM amplitude variability metrics, lead to an accurate identification of the CAP A phases with lower work time compared to visual CAP analysis [29]. Despite the promising premises, various aspects of the automatic detection implemented by Ferri et al. [29] still remain unexplored. For example, its reliability in pathological subjects and the topographic dimension of the detection itself. Indeed, Ferri [29] did not analyse the inter-channel reliability of its algorithm for the CAP A detection; this represents a limit of this study, since the generators of the two CAP A frequency components have different scalp localization [4]. In this prospective, the best agreement with the visual CAP analysis highlights the EEG derivation most appropriate for the automatic CAP analysis. Simple wearable devices, using only this derivation, could be developed in the future to record the EEG activity and to perform the consequent automatic CAP analysis in a comfortable manner. For this reason, our study investigated the reliability of Ferri’s algorithm for the correct and reliable recognition of the CAP A phases on several single derivations, in normal and in pathological subjects with different sleep disorders. Moreover, since the visual CAP A events are better detected considering multiple EEG channel analysis, multiple-trace algorithms of Ferri’s method were developed. In particular, a two-step strategy method was used: (a) adapted Ferri’s automatic method based on amplitude thresholds analysis in both the time and frequency domains was used in order to detect events with higher amplitude than the background voltage corresponding to CAP A phases in each EEG derivation analysed; (b) all CAP A events identified for each considered derivation were then compared and analysed with different types of multi-trace algorithms. In comparison with visual CAP analysis as the gold standard, true positive (TP), false negative (FN) and false positive (FP) were assessed in order to evaluate the reliability of the automatic detection methods both when single and multi-trace algorithms were used.

## Materials and methods

### Subjects

This study was performed on the EEG signals obtained from sleep recordings carried out on 41 subjects including healthy subjects (n = 8) as well as patients with different sleep disorders (n = 33, insomnia (n = 9), bruxism (n = 1), sleep-disordered breathing (n = 4), REM behavior disorder (n = 19)). The analyzed EEG recordings are publicly available on Physionet [35].

### Database

Single night polysomnographic recordings were exported from the CAP Sleep Database of Physionet [35] where, for each subject, the sleep macrostructure/microstructure scoring was available. Depending on the subjects, the EEG had different sampling rate, from 100 to 512 Hz and the EEG derivations used in this study were F2-F4/Fp2-F4, F4-C4, C4-P4, P4-O2, C4-A1, and F4-A1 as a result of the C4-A1 signal subtraction from F4-C4. These derivations were selected as they were available for the highest number of subjects, and they would guarantee a topographic analysis of the right hemisphere.

### Sleep quality assessment

Sleep onset was considered as the first epoch of any sleep stage. Parameters related to sleep quality such as Total Sleep Time (TST), Percent Sleep (%-Sleep, total sleep time from the sleep onset and the last awakening expressed in percentage), and Wakefulness After Sleep Onset (WASO) were assessed.

### Data pre-processing

EEG data were analysed via custom routines based on EEGLAB toolbox [36] and Matlab R2017b (MathWorks, Natick, MA, USA). Continuous EEG signals were band-pass filtered between 7 and 25 Hz (with the exclusion of the sigma frequency band by notch filter centered on 13 Hz with a bandwidth at 4 Hz) and between 0.3 and 4.5 Hz, in order to identify the fast and the slow components of the CAP A phases, respectively [29].

### Local analysis

**Fig 1:**
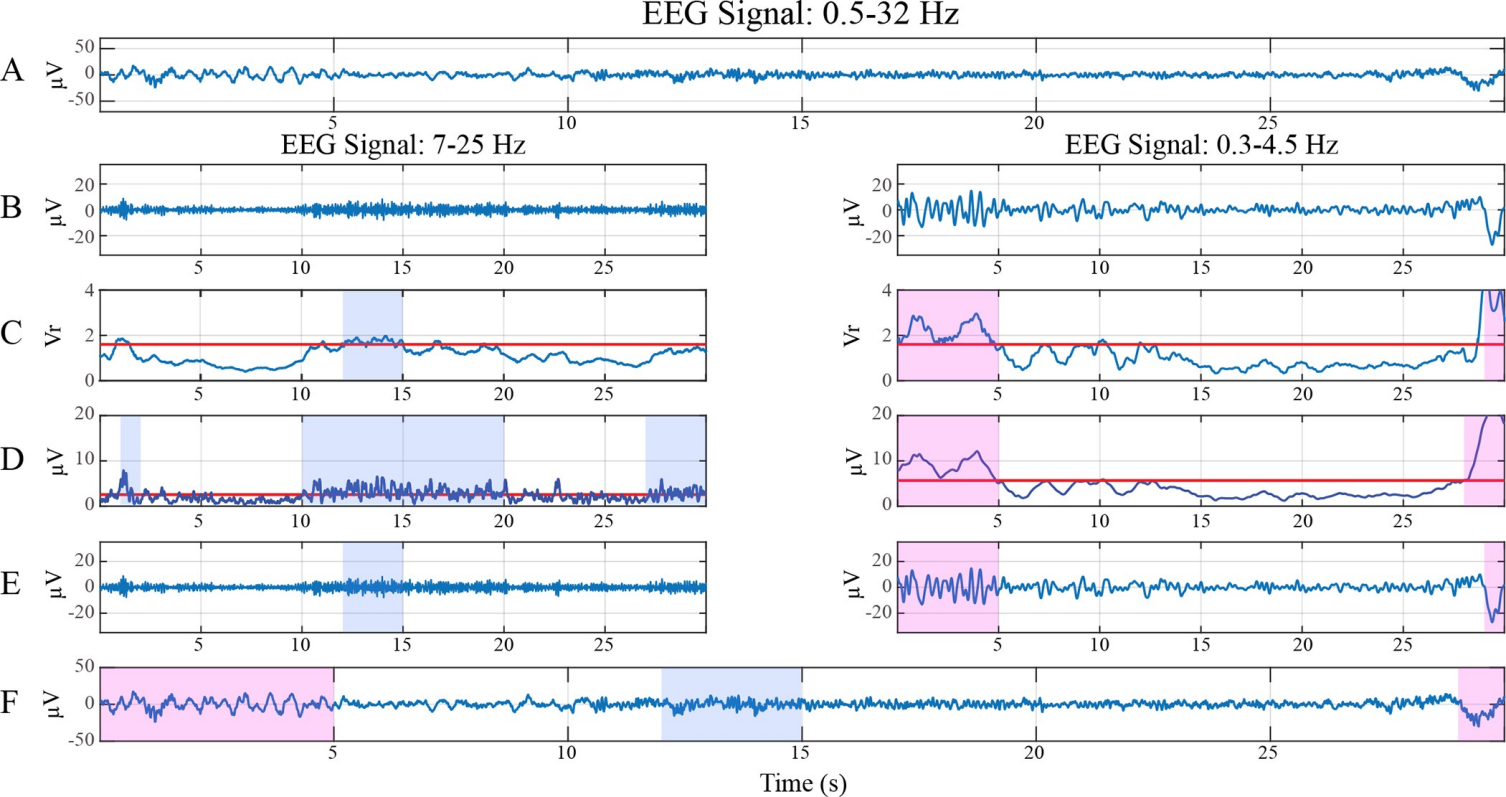
Graphic representation of the adapted Ferri’s algorithm. The raw EEG signal (A) was digitally band-pass filtered in the slow (B, right panel) and fast (B, left panel) frequency range (slow: 0.3–4.5 Hz; fast: 7–25 Hz). C) For each frequency band of interest, all seconds of the EEG signal with a variability measure 1.6 times (red line) higher than the variability of the corresponding 90-s epoch were marked. D) All seconds of the EEG signal with an envelope higher than the RMS threshold (red line) were marked. The common seconds identified in C and D represent the CAP A events (E), highlighted in (F) on the RAW EEG signal (pink frame: slow events; blue frame: fast events).

Adapted Ferri’s algorithm (2012) [29] was applied to NREM sleep stage for each derivation. This algorithm identified the CAP A phases, i.e. events with amplitudes higher background voltage. The processing steps involved are depicted in Fig 1. Briefly, the NREM EEG signal (Fig 1A), band-pass filtered for the two frequency bands of interest (fast: 7–25 Hz; slow: 0.3–4.5 Hz) (Fig 1B), was segmented in 90 s epochs shifting along the signal by 30 s windows. For each frequency band and for each such epoch, the root mean square (RMS) of the signal was computed and a threshold was set as 1.5xRMS. In the 30 central seconds of these 90 s epochs, the time stamps (in seconds) with a variability measure 1.6 times higher than the variability of the corresponding 90 s epoch were identified and marked (Fig 1B). Among these, only the time stamps during which the envelope of the filtered signal passed the RMS threshold (Fig 1C) were considered for the final step (Fig 1D). Indeed, at the end of this process, two different detection vectors were obtained, one for each frequency band of interest (7–25 Hz and 0.3–4.5 Hz), that were then recomputed in order to remove isolated events (events with only 1 s of duration) and to combine events separated by 1 s [1]. The obtained results highlighted the slow and the fast components of the CAP A phases that were finally merged together.

This adapted algorithm differed from the proposed Ferri’s method [29] in the evaluation of the RMS threshold. According to the CAP rules [1] that suggest the CAP A identification considering the surrounding background voltage, we decided to implement Ferri’s algorithm [29] evaluating the RMS threshold for each 90 s epoch and not for the entire NREM signal [29]. Indeed, applying Ferri’s algorithm and its adapted version to C4-A1 derivation, we observed that the NREM CAP time extracted using Ferri’s algorithm (109.27±61.44) was significantly lower than the NREM CAP time computed by the visual inspection (150.64±69.83, p≤0.005). Differently, no significant differences were found between the NREM CAP time based on visual scoring and the one automatically identified by the adapted Ferri’s algorithm (139.72±70.75). These observations suggest that the adapted Ferri’s algorithm is more reliable in the CAP analysis than the original Ferri’s method.

### Multi-trace analysis

**Fig 2:**
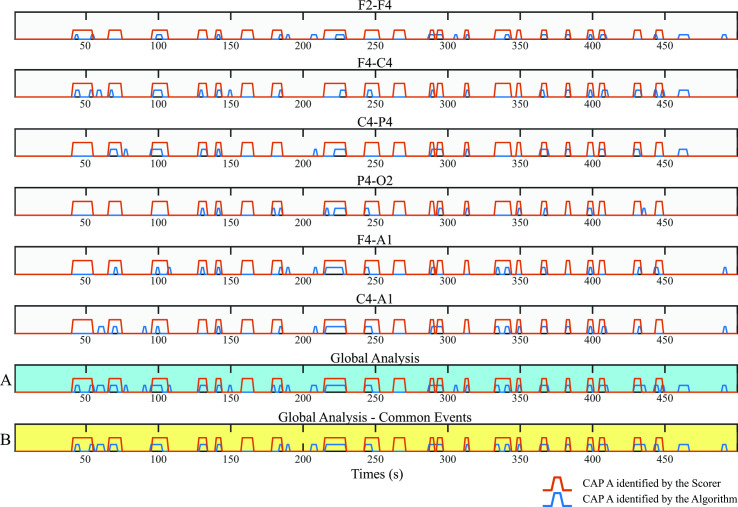
Graphic representation of the CAP A events identified by the algorithm (blue line) and by the scorer (red line). The first six rows from the top refer to a single analysed derivation (see row title). A and B represent the multi-trace approach. The raw with cyan background (A) represents the Global Analysis: all the events identified for each derivation analysed were pulled together and considered as CAP A definitive event. The raw with yellow background (B) represents the Global Analysis—Common Events.

From the CAP A events identified in each derivation, a multi-trace analysis was explored (Fig 2). In particular, the Global Analysis (Fig 2A) took into consideration all the events identified in each analysed derivation and, if two consecutive events were separated by 1 s interval, they were combined as a single event [1]. Moreover, another multi-trace algorithm was developed, here described as Global Analysis—Common Events (Fig 2B).

More specifically, the results of the Global analysis were followed by a rejection procedure: those CAP A events identified in only one out of six derivations were discarded, while the Common Events (occurring in at least two derivations simultaneously) were maintained to further comply with the scoring guidelines defining CAP as a “global EEG phenomenon involving extensive cortical areas. Therefore, phase As should be visible on all EEG leads. Bipolar derivations such as Fp1-F3, F3-C3, C3-P3, P3-O1 or Fp2-F4, F4-C4, C4-P4, P4-O2 guarantee a favorable detection of the phenomenon” [1].

### Performance metrics

To evaluate the performance of the adapted Ferri’s algorithm [29] on a single derivation and on the multi-trace algorithms, the TP (true positive: CAP A events marked by the expert and correctly detected by the algorithm), the FN (false negative, CAP A events marked by the expert but not detected by the algorithm), and the FP (false positive, the number of CAP A events detected by the algorithm but which were not marked by the expert) metrics were extracted using the visual scoring as the gold standard. The TP, FN, FP extraction permitted the computation of FNR (False Negative Rate, (FN/(FN+TP))*100), FDR (False Discovery Rate, (FP/(FP+TP))*100), Sensitivity ((TP/(TP+FN))*100), and Precision ((TP/(TP+FP))*100). The combination of these two latter metrics (Sensitivity and Precision) was instrumental for the extraction of F1 (accuracy measure, (2TP/(2TP+FP+FN))*100), the most compelling metrics available, as both high Sensitivity and high Precision are required for high F1 measure.

### CAP parameters

According to CAP’s rules [1], CAP sequences were detected and the subsequent CAP parameters extracted from the visual CAP scoring and from the CAP A automatically identified by the algorithms. The resulting standard CAP parameters were: NREM/LS/SWS CAP Time (min) and NREM/LS/SWS CAP Rate (%). Moreover, the total number of CAP A events and their mean duration (s) were evaluated.

### Statistical analysis

Statistical analysis was performed with Statistical Package for Social Sciences (SPSS, version 20) and the significance level was set at p≤0.05. A univariate analysis with the Independent t-test was used to detect significant differences between the normal and the pathological subjects. Paired t-test was used to identify differences in the performance metrics between each analysed derivation and between the local and the multi-trace algorithms. Moreover, differences in CAP A total number and their mean duration between the algorithm detection and the scorer were analysed by paired t-test. The relations between the standard CAP parameters obtained by the algorithm and by the scorer were tested by Pearson’s correlations.

## Results

### Sleep quality: Differences between normal and pathological subjects

Descriptive statistics of sleep quality measures (TST, WASO, %-Sleep) of the normal and pathological analysed subjects is summarized in Table 2. Univariate analysis identified significant differences in sleep quality metrics between the normal and the pathological subjects: pathological subjects showed higher WASO and lower TST and %-Sleep than the normal subjects (Table 2).

**Table 2:** Sleep quality parameters.

	Whole Sample (n = 41)	A. Normal Subjects (n = 8)	B. Pathological Subjects (n = 33)	A vs B
				p =
**TST (min)**	392.57±91.58	462.19±57.28	375.70±90.88	0.015
**WASO (min)**	67.65±51.80	18.56±21.48	79.55±55.04	0.002
**%-Sleep (%)**	84.96±12.03	96.15±4.40	82.25±11.74	0.002

Descriptive statistics (Mean±SD) of the sleep quality measures (TST, WASO, %-Sleep) in the normal (A) and pathological (B) subjects and comparison of the different variables using independent sample t-test.

### Performance of the automatic method using the local and the multi-trace approaches

For each derivation and for each multi-trace algorithm, the performance of the adapted Ferri’s algorithm in the CAP A events recognition was evaluated with respect to the visual CAP analysis used as the gold standard.

#### Local analysis

In the whole sample, F4-C4 derivation showed higher Sensitivity (71.13±12.53) and Accuracy (F1: 63.39±9.10) than the other derivations, with no significant differences in Sensitivity as compared to F2-F4/Fp2-F4 (Sensitivity: 70.13±12.22). Univariate analysis did not reveal significant differences in the algorithm performance between the normal and the pathological subjects (for F1 performance metric, see Table 3). The only exception is for P4-O2 Sensitivity, higher in normal as compared to pathological subjects. As observed in the whole sample, both subgroups showed significantly higher algorithm Sensitivity in F4-C4 than in C4-P4, P4-O2, F4-A1 and C4-A1, both in normal and in pathological subjects. Accuracy (F1) in F4-C4 was significantly higher than all the other analysed derivations in the pathological subjects. Moreover, FDR and Precision in F4-C4 were significantly different than in C4-P4/P4-O2 and in F2-F4/Fp2-F4 in normal and pathological subjects, respectively.

**Table 3:** F1—performance metric.

	Derivation	Whole Sample (n = 41)	A. Normal Subjects (n = 8)	B. Pathological Subjects (n = 33)	A vs B
					p =
**F1 (%)**	F2-F4/Fp2-F4	61.41±8.73	59.56±5.20	61.86±9.40	NS
	F4-C4	63.39±9.10	61.38±8.33	63.88±9.34	NS
	C4-P4	61.17±9.99	62.77±10.71	60.78±9.95	NS
	P4-O2	54.98±10.10	58.92±6.91	54.03±10.60	NS
	F4-A1	57.97±8.50	59.79±4.26	57.53±9.23	NS
	C4-A1	58.78±8.66	60.52±6.18	58.36±9.19	NS

Descriptive statistics (Mean±SD) of the algorithm’s F1 performance metric obtained in each derivation analysed for the whole sample and for the normal (A)/pathological (B) subjects. Independent sample t-test was conducted in all the different variables.

#### Multi-trace approach

In the whole sample and in each subgroup, both multi-trace methods (Global Analysis and Global Analysis—Common Events) led to a significant improvement in Sensitivity (Table 4), as compared to the best single derivation detection (F4-C4).

**Table 4:** Performance metrics: Local and multi-trace algorithms.

		1. F4-C4	2. Global Analysis	3. Global Analysis—Common Events	1 vs 2	2 vs 3	1 vs 3
					p =	p =	p =
**A. Whole Sample (n = 41)**	**Sensitivity (%)**	71.13±12.53	91.96±5.84	82.68±9.15	0.000	0.000	0.000
	**FNR (%)**	28.87±12.53	8.04±5.84	17.32±9.15	0.000	0.000	0.000
	**FDR (%)**	40.11±13.97	55.75±13.11	42.88±12.66	0.000	0.000	0.000
	**Precision (%)**	59.89±13.97	44.24±13.11	57.12±12.66	0.000	0.000	0.000
	**F1 (%)**	63.39±9.10	58.40±10.68	66.31±8.08	0.000	0.000	0.000
**B. Normal Subjects (n = 8)**	**Sensitivity (%)**	75.23±10.59	95.09±3.01	87.41±7.04	0.000	0.002	0.000
	**FNR (%)**	24.77±10.59	4.91±3.02	12.60±7.04	0.000	0.002	0.000
	**FDR (%)**	47.68±8.38	62.43±6.51	48.71±6.69	0.000	0.000	NS
	**Precision (%)**	52.32±8.38	37.57±6.51	51.29±6.69	0.000	0.000	NS
	**F1 (%)**	61.38±8.33	53.59±6.86	64.34±5.81	0.001	0.000	NS
**C. Pathological Subjects (n = 33)**	**Sensitivity (%)**	70.14±12.91	91.20±6.14	81.54±9.32	0.000	0.000	0.000
	**FNR (%)**	29.86±12.90	8.80±6.14	18.46±9.32	0.000	0.000	0.000
	**FDR (%)**	38.27±14.52	54.13±13.84	41.47±13.42	0.000	0.000	0.000
	**Precision (%)**	61.73±14.52	45.87±13.84	58.53±13.42	0.000	0.000	0.000
	**F1 (%)**	63.88±9.34	59.57±11.19	66.78±8.54	0.001	0.000	0.000

Descriptive statistics (Mean±SD) of algorithm’s performance metrics obtained in F4-C4 and with the multi-trace algorithms in whole sample (A), normal (B) and pathological (C) subjects and comparison of the different variables. Significance was tested by paired t-tests (Columns: 1 vs 2, 2 vs 3, 1 vs 3).

This improvement in Sensitivity occurred at the expense of Precision and FDR. In fact, in comparison with the F4-C4 detection, Precision decreased while FDR increased for both multi-trace algorithms. Nevertheless, in the Global Analysis—Common Events method, we observed a significant increment of the Accuracy (F1) as compared to F4-C4 (Table 4), suggesting this approach as the best algorithm for the CAP A automatic detection. Moreover, regardless of analysis type (local: F4-C4; multi-trace: Global Analysis and Global Analysis–Common Events), no significant differences were found in Sensitivity, FNR, FDR, Precision and F1 between normal and pathological subjects.

### Comparison between CAP visual scoring and automatic detection algorithms

As indicated above, F4-C4 and Global Analysis—Common Events are the best performing algorithms for the detection of correct CAP A, the first as compared to other single derivation algorithms, the second as compared to the other multi-trace proposed algorithm (Global Analysis). For these two algorithms, a further comparison between the CAP parameters (such as CAP rate and CAP time) based on visual scoring and the ones automatically identified by the algorithms was performed.

**Fig 3:**
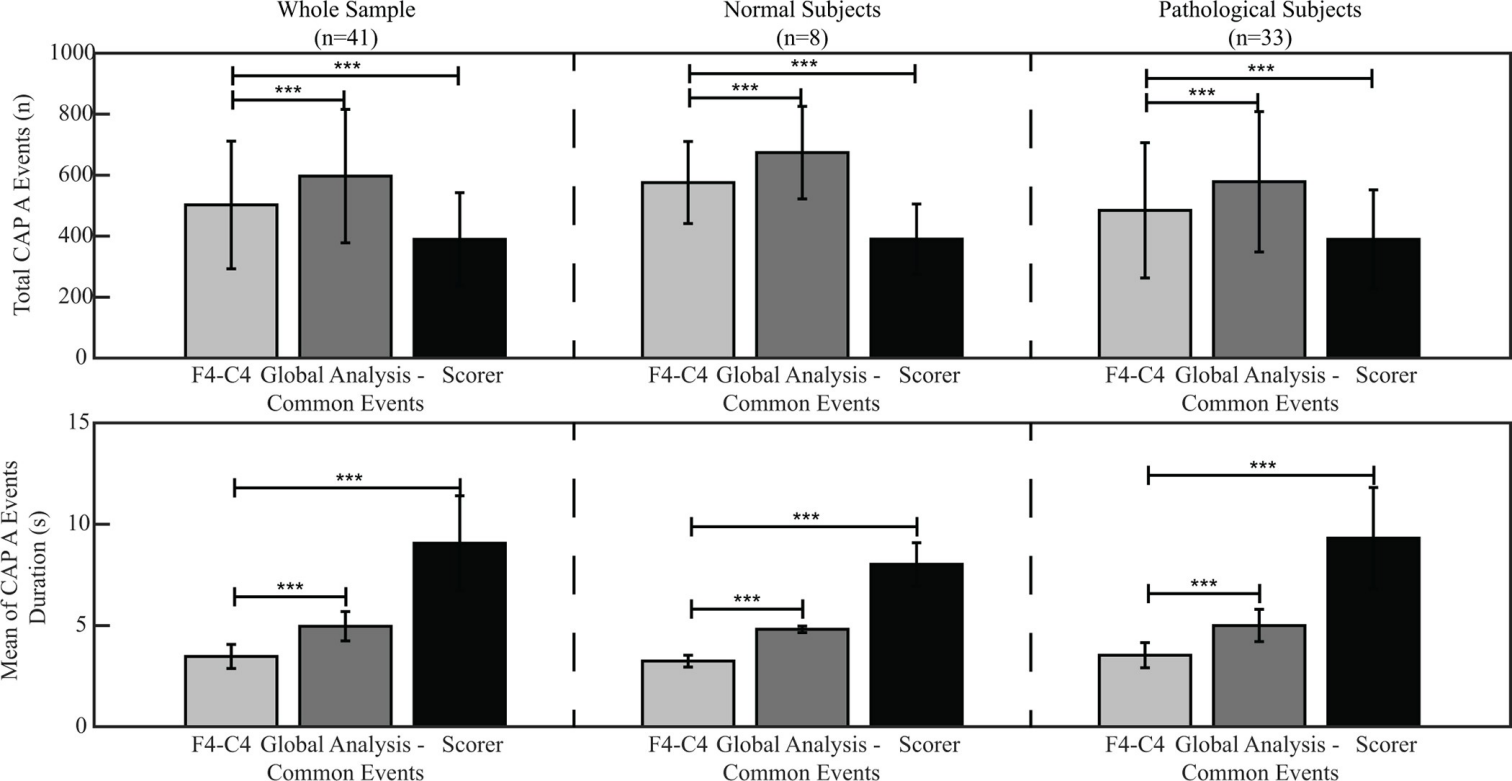
Graphic representation of the total CAP A with their mean duration detected by the algorithms (F4-C4 and global analysis–common events) and by the scorer in the whole sample, as well as in the normal and pathological subjects. Paired t-test was used for the comparisons. Asterisks indicate significant differences (*, p≤0.05; **, p≤0.01; ***, p≤0.005).

In the whole sample, as well as in normal and pathological subjects, the CAP A detected by these two algorithms were more numerous and with shorter mean duration than the ones identified by the scorer (Fig 3).

Nevertheless, significant good correlations were found between the standard CAP parameters based on visual scoring and the ones automatically identified by the algorithms (Table 5).

**Table 5:** Pearson’s correlations.

		Whole Sample (n = 41)		Normal Subjects (n = 8)		Pathological Subjects (n = 33)	
Algorithm	Scorer	F4-C4	Global Analysis—Common Events	F4-C4	Global Analysis—Common Events	F4-C4	Global Analysis—Common Events
**NREM CAP Time (min)**	**NREM CAP Time (min)**	R = 0.714***	R = 0.751***	R = 0.859**	R = 0.890***	R = 0.730***	R = 0.780***
**NREM CAP Rate (%)**	**NREM CAP Rate (%)**	R = 0.452,***	R = 0.570***	NS	NS	R = 0.493***	R = 0.614***
**LS CAP Time (min)**	**LS CAP Time (min)**	R = 0.728***	R = 0.783***	R = 0.894***	R = 0.937***	R = 0.768***	R = 0.827***
**LS CAP Rate (%)**	**LS CAP Rate (%)**	R = 0.365*	R = 0.512***	NS	R = 0.719*	R = 0.450**	R = 0.557***
**SWS CAP Time (min)**	**SWS CAP Time (min)**	R = 0.844***	R = 0.886***	R = 0.825*	R = 0.941***	R = 0.840***	R = 0.868***
**SWS CAP Rate (%)**	**SWS CAP Rate (%)**	R = 0.502***	R = 0.597***	NS	R = 0.732*	R = 0.437*	R = 0.549***

F4-C4 and Global Analysis—Common Events: Pearson’s correlations between the standard CAP parameters based on visual scoring and the ones automatically identified by the algorithms in the whole sample and in both normal and pathological subjects. Asterisks refer to significant correlations (*, p≤0.05; **, p≤0.01; ***, p≤0.005).

## Discussion

The primary aim of the present study was to further contribute to the development of an automatic tool for cyclic alternating pattern (CAP) detection, an established marker of sleep stability. The use of CAP in clinical settings has been limited by the time-consuming procedures necessary for its implementation. The aims of this paper were manifold: first, to identify the most reliable algorithm developed so far. Second, to describe its limitations. Third, to try to improve it in the perspective of a clinical application. Ferri’s algorithm [29] has several advantages: among others, it is parametric and very well described in each processing step and it reflects the originally proposed visual scoring criteria. A first methodological aspect that we explored hereby is the topographic dimension of the analysis. Indeed, Ferri and colleagues developed this automatic method focusing only on the C4-A1 derivation of healthy subjects [29]. We investigated the reliability of the same algorithm on several single EEG leads in order to identify the derivation with the best performance as compared to the gold standard, i.e. visual CAP scoring. Another unexplored dimension of the aforementioned algorithm was its performance in pathological subjects with compromised sleep stability. To this aim, we extended the analysis to an overall population of 41 subjects available on the Physionet Database [35], including 8 normal and 33 pathological subjects, affected by a wide range of sleep disorders.

In particular, being the CAP a global EEG phenomenon involving extensive cortical areas [1], the A phases should be visible in an independent manner on different EEG leads and we found that the most favourable detection site was not at the C4-A1 derivation, but it was at F4-C4 bipolar derivation with significantly higher Sensitivity and Accuracy (F1) in comparison with the visual CAP A scoring. Moreover, good correlations were found between the standard CAP parameters (CAP time and CAP rate) based on the visual scoring and the ones automatically identified by the F4-C4 local analysis. In particular, the CAP rate correlations in the whole sample were in general weak and they were absent in the normal subgroup, probably due to the limited number of these subjects. Therefore, if one derivation only is to be selected, F4-C4 appears to be the best out of the investigated ones.

The F4-C4 best performance is in line with the knowledge that the A1 CAP subtype, representing the frontal activity [4], is the predominant form of all the A phases identified during the all NREM sleep stage and, in particular, during the descending branch of the sleep cycles [37]. These observations could have an important clinical approach: independently of a known pathological condition of the subject, the lean acquisition of F4-C4 EEG recording during an entire night of EEG sleep will permit the identification of the CAP A phases with good performance in a comfortable, simple, and minimally intrusive manner.

If F4-C4 derivation showed the best comparative performance (F1 (%): 63.39±9.10), the FDR and FNR were still relatively high (FNR (%): 28.87±12.53; FDR (%): 40.11±13.97). For these reason, we implemented, according to Terzano’s CAP scoring rules [1], an approach that would take into consideration the global aspect of CAP using multi-trace algorithms development, in the attempt to further optimize the detection, when multiple leads are available.

Our results show that the Sensitivity obtained with the multi-trace algorithms was always higher than in the F4-C4 only and, as expected, also a significant increment of the FDR, metrics related to an increment of the FPs identified. These latter could represent artefacts, that generally occur in one derivation or may be randomly distributed among derivations without recognizable and reproducible patterns of propagation [38]. Consistently, the Global Analysis detects more FPs as compared to Global Analysis—Common Events, as the latter overcomes both the local and the Global Analysis limitations related to artefacts identification. In fact, in the Global Analysis—Common Events, the exclusion from the Global Analysis results of the characteristic single derivation events (i.e. artefacts) led to a relevant FDR decrement with a significant improvement of Accuracy (F1). This improvement in Accuracy observed in the Global Analysis—Common Events was also evident respect to the local F4-C4 analysis. This evidence is in line with the observation that the correlations observed between the standard CAP parameters based on visual scoring and the ones automatically identified by the algorithms showed higher correlation coefficient in Global Analysis—Common Events as compared to F4-C4. At present our methods are not addressed to classify the detected CAP A phases since the CAP parameters clinically used are independent from this type of classification.

Despite that, an important limitation of this study is the use of visual analysis as the gold standard for assessing the performance metrics: in fact, visual CAP scoring is a complex process that requires deep expertise and arbitrary visual evaluation of amplitude differences from the specialists in sleep medicine. For this reason, we cannot exclude that automatic methods might be more precise than the visual CAP scoring in applying the detection rules, as previously suggested [29]. Indeed, the CAP A events identified by the algorithm on a single derivation and also on the Global Analysis—Common Events are more numerous and with shorter mean duration than the CAP A phases identified by visual inspection. For this reason, it might be interesting to evaluate whether the FP identified by the local or the multi-trace algorithms have really to be considered as such or are CAP A events not properly identified by the scorer during the NREM EEG visual inspection. An interesting further step would imply a visual supervision of the automatically identified events verifying if the expert scorer, guided by the automatic detection, changes his mind in the CAP A phases identification.

## Conclusion

This study aimed at further developing one of the best established algorithms in the automatic detection of CAP, by specifically exploring the best topographic location for the most accurate performance and, in parallel, the contribution of concurrent detections at different leads. Our results showed that F4-C4 is the best EEG derivation to be used for the proposed automatic CAP detection when a minimally invasive approach is necessary or preferable. When more EEG leads are available, instead, the proposed Global Analysis—Common Events represents a reliable method for measuring sleep instability. In both cases FNR and FDR are relatively high when compared to the manual/visual CAP scoring. We speculate that the automatic detection might be more precise in identifying systematically and objectively CAP and therefore the actual relevance of false detections is arguable. Moreover, the proposed algorithms have a low computational cost and are therefore ready to be implemented in a mono- or multi-channel embedded hardware device which, recording and processing EEG sleep data, will be able to identify directly the CAP A phases and extract the CAP parameters in an automatic and reliable manner. This system could support sleep clinicians in their daily routine helping to carefully monitoring sleep stability in a sustainable manner.
