# Correction: Automatic Cyclic Alternating Pattern (CAP) analysis: Local and multi-trace approaches

**DOI:** 10.1371/journal.pone.0316113

**Published:** 2024-12-17

**Authors:** Maria Paola Tramonti Fantozzi, Ugo Faraguna, Adrien Ugon, Gastone Ciuti, Andrea Pinna

There are errors in the author affiliations. The correct affiliations are as follows:

Maria Paola Tramonti Fantozzi^1,2,3^, Ugo Faraguna^3,4^, Adrien Ugon^1,5^, Gastone Ciuti^2^, Andrea Pinna^1^

**1** Laboratoire d’Informatique de Paris 6, CNRS, Sorbonne Universite´, Paris, France, **2** The BioRobotics

Institute, Scuola Superiore Sant’Anna, Pontedera, Italy, **3** Department of Translational Research and of New Surgical and Medical Technologies, University of Pisa, Pisa, Italy, **4** Department of Developmental

Neuroscience, IRCCS Fondazione Stella Maris, Pisa, Italy, **5** ESIEE-Paris, Cite´ Descartes, Noisy-le-Grand, France
